# The Impact of Surgery on Long-Term Survival of Patients with Primary Gastric Diffuse Large B-Cell Lymphoma: A SEER Population-Based Study

**DOI:** 10.1155/2019/9683298

**Published:** 2019-02-24

**Authors:** Ju-Li Lin, Jian-Xian Lin, Ping Li, Jian-Wei Xie, Jia-bin Wang, Jun Lu, Qi-Yue Chen, Long-long Cao, Chao-Hui Zheng, Chang-Ming Huang

**Affiliations:** ^1^Department of Gastric Surgery, Fujian Medical University Union Hospital, Fuzhou, Fujian Province, China; ^2^Department of General Surgery, Fujian Medical University Union Hospital, Fuzhou, Fujian Province, China; ^3^Key Laboratory of Ministry of Education of Gastrointestinal Cancer, Fujian Medical University, Fuzhou, Fujian Province, China

## Abstract

**Background:**

The aim of this retrospective study was to compare the long-term survival of patients receiving conservative with surgical treatment to analyze the prognostic factors and the impact of surgery on oncological outcomes of patients with primary gastric diffuse large B-cell lymphoma.

**Methods:**

A total of 2647 patients diagnosed with primary gastric diffuse large B-cell lymphoma from 1998 to 2014 were extracted from SEER database. Propensity matching was performed to compare the clinicopathological characteristics of the two groups. Based on the recursive partitioning analysis, the patients were divided into three risk subgroups: low risk, intermediate risk, and high risk.

**Results:**

After propensity score matching, patient characteristics did not differ significantly between the two groups. The 5-year cancer-specific survival rates of the surgical group and the conservative treatment group were, respectively, 60% and 59.2% (*P* = 0.952) before propensity matching and 64.2% and 58.6% (*P* = 0.046) after propensity matching. According to the multivariate analysis, age, tumor stage, and chemotherapy and surgery were independent risk factors for long-term survival. The 5-year cancer-specific survival rates differed significantly between the low-risk, intermediate-risk, and high-risk patients (76.2% vs. 57.4% vs. 25.5%, respectively, *P* < 0.001). The 5-year cancer-specific survival rate of the surgical group was significantly higher than that of the conservative treatment group in the low-risk patients. However, it did not differ significantly in the intermediate-risk and high-risk patients (*P* > 0.05).

**Conclusions:**

A prognostic model was constructed based on the independent risk factors of age, tumor stage, and chemotherapy. The prognostic model indicated that low-risk patients (age < 75 years, stage I/II, with/without chemotherapy) undergoing surgical treatment may benefit from long-term survival, while intermediate- and high-risk patients (age ≥ 75 years, stage I/II, with/without chemotherapy or III/IV patients, with/without chemotherapy) gain no significant benefit from surgery.

## 1. Introduction

According to the World Health Organization (WHO) classification [1], the predominant histological subtypes of primary gastric lymphomas (PGLs) are marginal zone B-cell lymphoma of mucosa-associated lymphoid tissue (MALT) and diffuse large B-cell lymphoma (DLBCL). Chemotherapy is recommended as the first-line treatment for DLBCL according to the Japanese gastric cancer treatment guidelines 2010 (ver. 3) and the NCCN Guidelines. Surgery is recommended as an urgent treatment for patients presenting with severe perforation, bleeding, or obstruction and as palliative treatment [2, 3]. Several studies [4–7] have shown that surgery can improve the long-term survival of DLBCL patients. However, most of these studies are published before 2003. Since the addition of rituximab in the DLBCL [8, 9], the survival rate has been improved. And evidence-based medicine is lacking due to the low incidence of the disease and because only retrospective studies with small sample sizes are available. The impact of surgery on long-term survival of patients with different prognostic factors for primary gastric DLBCL had not been reported. The current study is a retrospective analysis with a large sample size and uses the propensity score matching method to reduce bias. The aim of this retrospective study was to compare the long-term survival of the conservative treatment group to that of the surgical group in order to analyze the prognostic factors and the impact of surgery on oncological outcomes of primary gastric DLBCL.

## 2. Methods

### 2.1. Patient Selection

A case listing session was created from the Surveillance, Epidemiology, and End Results (SEER) program using SEER^∗^Stat 8.2.1 (http://seer.cancer.gov/seerstat). A total of 2647 patients diagnosed with primary gastric DLBCL from 1998 to 2014 were extracted from the SEER database. Among these patients, 275 cases were treated surgically, and 2372 cases were treated conservatively. The inclusion criteria were as follows: patients diagnosed between January 1998 and December 2014; Ann Arbor [10] staging codes ([EOD] 10 - extent [1988-2003]; Collaborative Stage [CS] extension [2004+] stage; I and II, and stage III and IV ([ICD-O-3] topography); [pathological diagnosis of code], 16.0-16.9) ([ICD-O-3] 9680/3); the operation code (RX Summ--Surg Prim Site (1998+), 30-80); chemotherapy recode: chemotherapy code (yes, no/unk)); and radiotherapy (radiation recode) code. The exclusion criteria were as follows: patients without any treatment, Ann Arbor staging is unknown, patients younger than 18 years old, multiple tumors (first malignant primary indicator), not a tumor-related death (SEER “other cause of death” classification), and the death of patients within 30 days. For the validation using our dataset, 50 patients who were diagnosed with primary gastric DLBCL from January 2003 to June 2013 in our center were enrolled. The inclusion criteria were defined as follows: histologically proven gastric DLBCL. The exclusion criteria were defined as follows: a lack of a pathological diagnosis, did not receive treatment, or died in 1 month after diagnosis.

### 2.2. Statistical Analysis

Statistical analysis was performed using SPSS v22.0 for Windows (SPSS Inc., Chicago, IL) and R 3.4.0 (http://www.r-project.org/). Categorical variables were analyzed using the chi-square or Fisher's exact tests, whereas continuous variables were analyzed using either the unpaired Student's *t*-test or the Mann-Whitney *U* test. Cumulative survival rates were estimated using the Kaplan-Meier method and compared using the log-rank test. Multivariate analyses were performed by the Cox regression model to identify the independent risk factors for long-term survival. Two-sided *P* values less than 0.05 were considered to be significant. Propensity matching was performed in R 3.4.0 using the nearest neighbor matching and a caliper width of 0.02. Recursive partitioning analysis provides a simple way to group patients into different categories. The R software with the rpart package was used for this analysis, which requires a minimum of 20 observations to split a node [11]. Recursive partitioning analysis can divide patients objectively into two groups based on the 5-year cancer-specific survival rate. This provides maximum survival discrimination and yields subgroups with relatively homogeneous survival performance. Based on the recursive partitioning analysis, patients were finally divided into three risk subgroups: low risk, intermediate risk, and high risk.

## 3. Results

### 3.1. Baseline Patient Characteristics

The clinicopathological characteristics of the surgical group and the conservative treatment group are shown in Table 1. Before propensity score matching, there were 275 patients in the surgical group and 2372 patients in the conservative treatment group. Age, gender, and race did not differ significantly between the two groups, while tumor location, cancer stage, and chemotherapy and radiotherapy differed significantly between the two groups. The propensity scores were calculated using a logistic regression model to balance the following covariates: tumor location, cancer stage, and chemotherapy and radiotherapy. Finally, 1890 patients (210 patients in the surgical group and 1680 patients in the conservative treatment group) were selected for analysis. After propensity score matching, patient characteristics were not observed to differ significantly between the two groups (*P* > 0.05).

### 3.2. Long-Term Survival Analysis

**Figure 1:**
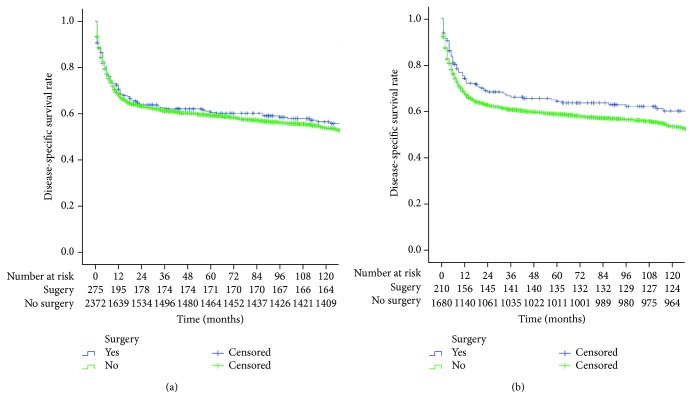
(a) Comparison of 5-year CSS between the surgical and conservative treatment groups before propensity matching (*P* = 0.952); (b) comparison of 5-year CSS between the surgical and conservative treatment groups after propensity matching (*P* = 0.0462).

The 5-year cancer-specific survival rate (CSS) of all patients was 59.3%. The 5-year CSS of the surgical group and the conservative treatment group were 60% and 59.2% (*P* = 0.952), respectively, before propensity matching, as shown in Figure 1(a). After propensity matching, the 5-year CSS of the surgical group and the conservative treatment group were 64.2% and 58.6% (*P* = 0.046), respectively, as shown in Figure 1(b).

### 3.3. Independent Risk Factors for Long-Term Survival

A univariate analysis showed that age, gender, tumor stage, and chemotherapy, radiotherapy, and surgery (*P* < 0.05) were closely related to long-term survival. According to the multivariate analysis, age, tumor stage, and chemotherapy and surgery were independent risk factors for long-term survival (Table 2).

### 3.4. Prognostic Model Based on Recursive Partitioning Analysis

**Figure 2:**
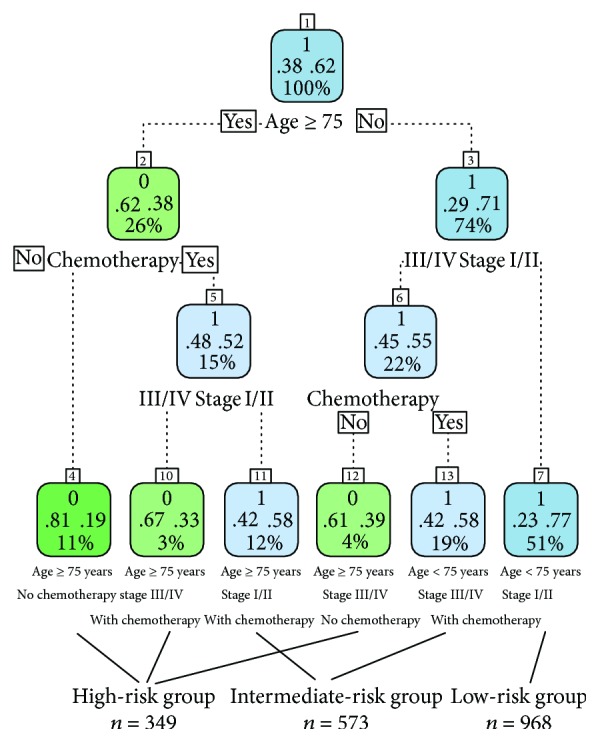
After propensity score matching, 1890 patients were selected for recursive partitioning analysis. Low-risk patients: 51% (age < 75 years, stage I/II, with/without chemotherapy), intermediate-risk patients: 31% (age ≥ 75 years, stage I/II patients, chemotherapy; age < 75, stage III/IV, with chemotherapy), and high-risk patients: 18% (age ≥ 75 years, without chemotherapy; age ≥ 75 years, stage III/IV, with chemotherapy; age < 75 years, stage III/IV, without chemotherapy) (number 0 means survival rate < 50% and number 1 means survival rate ≥ 50%). For example: 1, .38, .62, and 100% in the 1st box, the number 1 means survival rate ≥ 50%, .38 means the death rate is 38%, .62 means the survival rate is 62%, and 100% means the whole patients in the 1st box.

Recursive partitioning analysis provides a simple way to group patients into different categories. The analysis identified three predictors (age, tumor stage, and previous chemotherapy) according to the end point (5-year CSS) and the cutoffs that maximized the stratification for risk-specific survival. Patients aged ≥75 years had a worse 5-year CSS (37.62%) compared to patients aged <75 years (70.29%). We combined nodes with similar survival rates into the intermediate- and high-risk groups. Finally, the patients were ultimately divided into the following risk groups (Figure 2): low risk (age < 75 years, stage I/II, with/without chemotherapy), intermediate risk (age ≥ 75 years, stage I/II, with chemotherapy; age < 75 years, stage III/IV, with chemotherapy), and high risk (age ≥ 75 years, without chemotherapy; age ≥ 75 years, stage III/IV, with chemotherapy; age < 75 years, stage III/IV, without chemotherapy).

### 3.5. Stratified Survival Analysis

**Figure 3:**
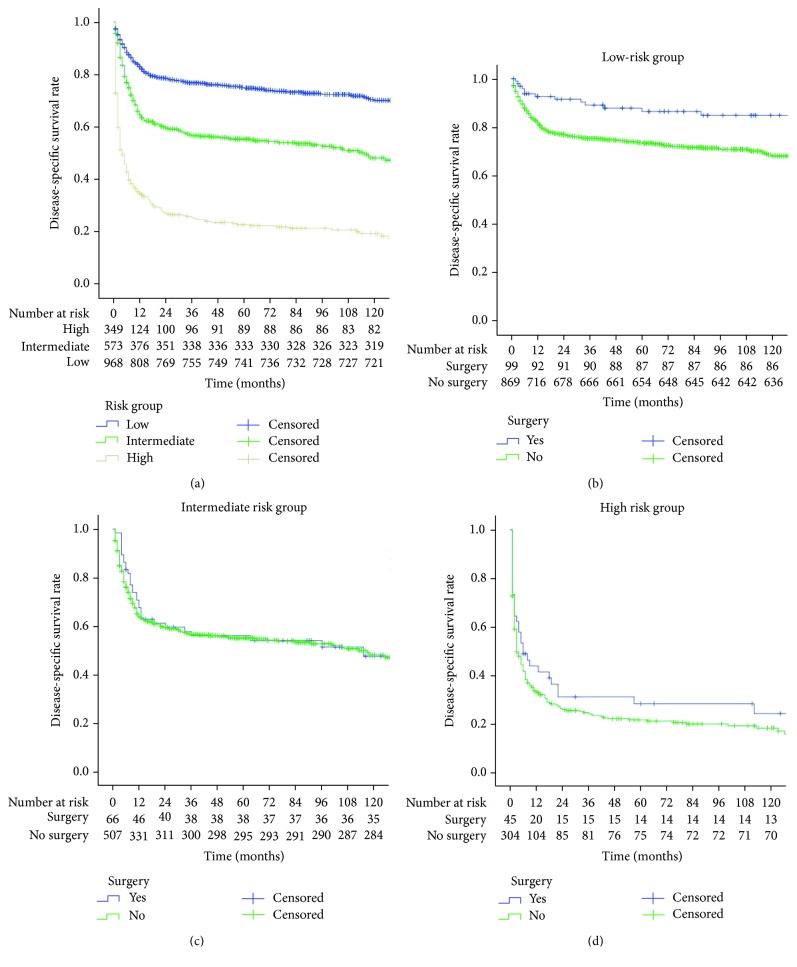
(a) There were statistically significant survival differences between the low-, intermediate-, and high-risk patients (*P* < 0.001); (b) there was a statistically significant survival difference between the surgical and conservative treatment groups in low-risk patients (*P* < 0.001); (c) there was no statistically significant survival difference between the surgical and conservative treatment groups in intermediate-risk patients (*P* = 0.737); (d) there was no statistically significant survival difference between the surgical and conservative treatment groups in high-risk patients (*P* = 0.199).

The 5-year CSS significantly differed between the low-risk, intermediate-risk, and high-risk patients (76.2% vs. 57.4% vs. 25.5%, respectively, *P* < 0.001, Figure 3(a)). The 5-year CSS of the surgical group and the conservative treatment group were 86.5% and 73.4% (*P* < 0.001), respectively, in the low-risk patients (Figure 3(b)). The 5-year CSS of the surgical group and the conservative treatment group did not differ significantly in the intermediate- and high-risk patients (Figures 3(c) and 3(d)).

### 3.6. Extracted Validation by Using our Dataset

The clinicopathological characteristics of our center are shown in Supplement Table 1. The follow-up time ranged from 9 to 144 months, and the mean follow-up time is 54.7 months. 42 patients of our center received both surgery and chemotherapy, 7 patients only received chemotherapy, and 1 patient only received surgery. The 5-year CSS of patients in the surgical group was 78.8%, while the 5-year CSS of patients in the conservative treatment group was 68.6% (Supplement Figure 1a). The 5-year CSS of patients in the surgical group (86.4%) is higher than the 5-year CSS of patients in the conservative treatment group (66.7%) in low-risk patients (Supplement Figure 1b).

## 4. Discussion

Primary gastric lymphoma (PGL) is a rare tumor, accounting for 2% to 8% of all gastric neoplasms [12, 13]. DLBCL represents 45%-50% of all primary gastric lymphomas (PGLs). Only a small number of studies have investigated the long-term survival rates of patients and primarily examined small cohorts of patients due to the low incidence of the disease. Currently, the role of surgery on the survival of DLBCL patients with different prognostic factors had not been reported. Therefore, our study investigated a large number of patients to analyze the clinical characteristics, prognostic factors, and role of surgery on the survival of DLBCL patients. Specifically, the propensity score matching method was used to reduce bias [14]. Before propensity score matching, tumor location, tumor stage, and previous radiotherapy and chemotherapy were observed to differ significantly between the surgical group and the conservative treatment group. After propensity score matching, baseline patient characteristics did not differ significantly between the two groups. To the best of our knowledge, the current study has enrolled the largest number of patients with DLBCL to date, and the results provide new direction for conducting randomized controlled clinical trials in the future.

Propensity score matching (PSM) [14] is a statistical matching technique that attempts to estimate the effect of a treatment, policy, or other intervention by accounting for the covariates that predict receiving the treatment. PSM attempts to reduce the bias due to confounding variables that could be found in an estimate of the treatment effect obtained from simply comparing outcomes among units that received the treatment versus those that did not. In the current study, we found that there were some clinicopathological characteristics that were different between the surgery and no surgery groups. On the other hand, in order to include more patients, we used one to eight matching analysis. After PSM, these differences disappeared between the surgery and no surgery groups.

Prognostic factors of DLBCL are not well-defined. Koch et al. [15] reported that the survival rate at 42 months for patients treated with chemotherapy combined with radiotherapy was 91.0%. The NCCN Guidelines recommended radiotherapy when the diameter of the tumor is more than 7.5 cm after 6 cycles of RCHOP [3]. In this study, previous radiotherapy was closely related to long-term survival according to univariate analysis, but multivariate analysis did not show a significant association. According to the multivariate analysis, age, tumor stage, and chemotherapy and surgery were independent risk factors for long-term survival. Because of overall poor functionality, low resilience, and short life expectancy, the survival rate of elderly patients is relatively low. Therefore, we should pay special attention to the management of elderly patients and establish appropriate treatment programs to reduce the incidence of postoperative complications. Previous studies [7, 16, 17] suggested that tumor stage is an important risk factor for long-term survival, and the survival rate of early stage patients is higher than that of patients with advanced stage tumors. Chemotherapy is one of the main treatment methods for primary gastric DLBCL. This treatment plays an important role in the prognosis of patients, and chemotherapy protocols should be individualized to each patient. Avilés et al. [18] reported that in 150 patients with stage I/II tumors who received CHOP, the 10-year event-free survival (EFS) was 96%. Sohn et al. [8] found that out of 38 patients who received CHOP and 55 patients who received R-CHOP, the 3-year overall survival rates were 95% and 85% (*P* > 0.05), respectively. Some previous studies [18] showed that for localized disease of DLBCL of stomach, the short course of R-CHOP and the antibiotic treatment are demonstrated to provide a better event-free survival and CSS.

At present, the role of surgery in patients for primary gastric DLBCL remains controversial. Binn et al. [19] conducted a prospective study of 106 patients, and the results showed that chemotherapy combined with surgery did not have a statistically significant survival advantage over patients who received chemotherapy alone. A diagnosis of primary gastric DLBCL is usually established by the examination of a biopsy specimen obtained during an endoscopy. Missed detection and misdiagnosis are possible when the specimen is too small, and it can be difficult to obtain satisfactory histological specimens. The complete resection of the tumor is helpful for histopathological examination and correct staging. At the same time, resection of the lesion can reduce the tumor burden in vivo and improve postoperative radiotherapy and chemotherapy efficacy. Barbara et al. [6] reported a single center retrospective study with 106 patients who received surgery combined with chemotherapy and 39 patients who received chemotherapy alone and observed that the 5-year overall survival rates were 97.2% and 89.2% (*P* = 0.046), respectively. The study by Wang et al. [7] showed that out of 75 patients who received surgery combined with chemotherapy and 33 patients who received chemotherapy alone, the 5-year overall survival rates were 80.1% and 49.8% (*P* = 0.046), respectively. Combining the results of the above studies, we hypothesized that surgery has an important impact on the prognosis of primary gastric DLBCL.

However, there are no reports regarding the impact of surgery on long-term survival of patients with different prognostic factors for primary gastric DLBCL. To guide clinical practice, individualized treatment strategies should be adopted for patients depending on risk factors. We used the recursive partitioning analysis to construct the prognostic models of patients with primary gastric DLBCL [20–22]. The recursive partitioning analysis method establishes a decision tree based on the independent risk factors that affect the survival rate, merging nodes with similar survival rates. Ultimately, risk stratification was performed with the goal to guide individualized treatment for each patient. The model is accurate, easy to understand, and suitable for generalization and clinical decision making. The decision tree model was established based on clinicopathological characteristics, such as age, tumor stage, and chemotherapy. Lastly, patients were divided into high-risk, intermediate-risk, and low-risk groups according to the end point (5-year CSS). Survival analysis showed that the long-term survival rate of low-risk patients was significantly higher than that of intermediate-risk and high-risk patients. Stratified analysis was performed on patients with different risk factors; low-risk patients can benefit from surgical treatment for long-term survival, while intermediate- and high-risk patients gain no significant benefit from surgery.

The 5-year CSS of patients in the surgical group of our center is 78.8%, similar to the survival rate of patients undergoing surgery reported in previous studies [6, 7] (80.1%-97.2%). And the 5-year CSS of patients in the surgical group (86.4%) is higher than the 5-year CSS of patients in the conservative treatment group (66.7%) in the low-risk group. Because of the limited sample size, it is not possible to further analyze the prognosis of patients with chemotherapy alone vs. surgery alone vs. surgery plus chemotherapy in the low-risk group. Further analysis is needed in the future.

This study investigated a large number of patients with primary gastric DLBCL and used the propensity score matching method to reduce bias. However, our study is subject to several limitations. We conducted the study in a retrospective manner, and there was data selection bias. The SEER database is incomplete; many important clinical characteristics, such as details regarding chemotherapy, radiotherapy, histological evidence of MALT in gastric DLBCL, postoperative complications, ECOG, serum LDH, and recurrence, were not included. Although it has been reported that LDH is one of the important prognostic factors of DLBCL, this study cannot further analyze the effect of LDH on the prognosis of DLBCL due to SEER database limitation. And it is also hard to analyze histological evidence of MALT in gastric DLBCL. And we can do more research on the analysis of LDH and histological evidence of MALT in gastric DLBCL in further study.

In conclusion, our study investigated a large number of patients and analyzed clinical characteristics and prognostic factors. We observed that age, tumor stage, and previous chemotherapy and surgery were independent risk factors for long-term survival. Specifically, the propensity score matching method was used to reduce bias. After propensity score matching, the 5-year CSS of the surgical group was determined to be higher than that of the conservative treatment group. Furthermore, a prognostic model based on recursive partitioning analysis was established for the first time. The prognostic model indicated that low-risk patients (age < 75 years, stage I/II, with/without chemotherapy) who undergo surgical treatment can benefit from an improved survival rate, while intermediate- and high-risk patients (age ≥ 75 years, stage I/II, with/without chemotherapy or III/IV patients, with/without chemotherapy) gain no significant benefit from surgery. Additional randomized controlled clinical trials should be conducted to provide further evidence.

## Figures and Tables

**Table 1:** Baseline patient characteristics.

	Before matching			After matching		
Variable	Surgery (*n* = 275)	No surgery (*n* = 2372)	*P*	Surgery (*n* = 210)	No surgery (*n* = 1680)	*P*
Age			0.146			0.446
<65	127 (46.2%)	1113 (46.9%)		99 (47.1%)	848 (50.5%)	
65-74	75 (27.3%)	533 (22.5%)		48 (22.9%)	396 (23.6%)	
≤75	73 (26.5%)	726 (30.6%)		63 (30%)	436 (26%)	
Gender			0.308			0.575
Male	152 (55.3%)	1387 (58.5%)		118 (56.2%)	978 (58.2%)	
Female	123 (44.7%)	985 (41.5%)		92 (43.8%)	702 (41.8%)	
Race			0.088			0.331
White	223 (81.1%)	1815 (76.5%)		175 (83.3%)	1353 (80.5%)	
Other	52 (18.9%)	557 (23.5%)		35 (16.7%)	327 (19.5%)	
Tumor location			0.024			0.136
Upper third	20 (7.3%)	316 (13.3%)		18 (8.6%)	226 (13.5%)	
Mid and low third	115 (41.8%)	861 (36.3%)		86 (41%)	604 (36%)	
Overlapping stomach	33 (12%)	259 (10.9%)		28 (13.3%)	191 (11.4%)	
Unknown location	107 (38.9%)	936 (39.5%)		78 (37.1%)	659 (39.2%)	
Ann Arbor stage			<0.001			0.706
I	113 (41.1%)	1054 (44.4%)		102 (48.6%)	770 (45.8%)	
II	88 (32%)	494 (20.8%)		47 (22.4%)	436 (26%)	
III	12 (4.4%)	199 (8.4%)		9 (4.3%)	78 (4.6%)	
IV	62 (22.5%)	625 (26.3%)		52 (24.8%)	396 (23.6%)	
Radiation			<0.001			0.666
Yes	20 (7.3%)	482 (20.3%)		20 (9.5%)	145 (8.6%)	
No	255 (92.7%)	1890 (79.7%)		190 (90.5%)	1535 (91.4%)	
Chemotherapy			<0.001			0.894
Yes	175 (63.6%)	1900 (80.1%)		159 (75.7%)	1279 (76.1%)	
No/unknown	100 (36.4%)	472 (19.9%)		51 (24.3%)	401 (23.9%)	

**Table 2:** Independent risk factors of long-term survival after propensity score matching.

Variable	Univariate analysis *P*	Multivariate analysis			*P*
		HR	95% CI		
Age	<0.001				<0.001
<65		1(Ref)			
65-74		1.344	1.108	1.63	0.003
≤75		3.053	2.586	3.603	<0.001
Gender	0.009				0.919
Male		1(Ref)			
Female		1.007	0.873	1.162	
Race	0.918				
White					
Other					
Tumor location	0.147				
Upper third					
Mid and low third					
Overlapping stomach					
Unknown location					
Ann Arbor stage	<0.001				<0.001
I		1(Ref)			
II		1.515	1.258	1.824	<0.001
III		1.869	1.332	2.624	<0.001
IV		2.413	2.037	2.858	<0.001
Radiation	0.022				0.435
Yes		1(Ref)			
No		1.115	0.849	1.464	
Chemotherapy	<0.001				<0.001
Yes		1(Ref)			
No/unknown		2.109	1.801	2.469	
Surgery	0.046				<0.001
Yes		1(Ref)			
No		1.361	1.077	1.719	

## Data Availability

The data used to support the findings of this study are available from the corresponding author upon request.

## Supplementary Materials

Supplementary Materials Supplement Table 1: the patient's clinicopathological characteristics of our center are shown in Supplement Table 1. Between January 2003 and June 2013, 50 patients who were diagnosed with primary gastric DLBCL in our center were enrolled. 33 patients are male; 17 patients are female. The follow-up time ranged from 9 to 144 months, and the mean follow-up time is 54.7 months. 17 patients, 23 patients, 4 patients, and 6 patients are stage I, stage II, stage III, and stage IV, respectively. 42 patients of our center received both surgery and chemotherapy, 7 patients only received chemotherapy, and 1 patient only received surgery. 38 patients, 2 patients and 10 patients are low-risk, intermediate-risk, and high-risk patients, respectively. Supplement Figure 1: the 5-year CSS of patients in the surgical group was 78.8%, while the 5-year CSS of patients in the conservative treatment group was 68.6% (Supplement Figure 1a). The 5-year CSS of patients in the surgical group (86.4%) is higher than the 5-year CSS of patients in the conservative treatment group (66.7%) in low-risk patients (Supplement Figure 1b).
