# Supplementary material for: The Impact of Surgery on Long-Term Survival of Patients with Primary Gastric Diffuse Large B-Cell Lymphoma: A SEER Population-Based Study
Source: Gastroenterol Res Pract. 2019 Feb 24;2019:9683298. doi: 10.1155/2019/9683298 (PMC6409055; doi:10.1155/2019/9683298)
Supplement: Supplementary Materials — Supplement Table 1: the patient's clinicopathological characteristics of our center are shown in Supplement Table 1. Between January 2003 and June 2013, 50 patients who were diagnosed with primary gastric DLBCL in our center were enrolled. 33 patients are male; 17 patients are female. The follow-up time ranged from 9 to 144 months, and the mean follow-up time is 54.7 months. 17 patients, 23 patients, 4 patients, and 6 patients are stage I, stage II, stage III, and stage IV, respectively. 42 patients of our center received both surgery and chemotherapy, 7 patients only received chemotherapy, and 1 patient only received surgery. 38 patients, 2 patients and 10 patients are low-risk, intermediate-risk, and high-risk patients, respectively. Supplement Figure 1: the 5-year CSS of patients in the surgical group was 78.8%, while the 5-year CSS of patients in the conservative treatment group was 68.6% (Supplement Figure 1a). The 5-year CSS of patients in the surgical group (86.4%) is higher than the 5-year CSS of patients in the conservative treatment group (66.7%) in low-risk patients (Supplement Figure 1b). [file 9683298.f1.docx]

**Supplement Table 1**

| Characteristics | Number of patients (%) |
| --- | --- |
| Age |  |
| 65 < | 41(82.0%) |
| 65-74 | 5(10.0%) |
| ≤75 | 4(8.0%) |
| Gender |  |
| Male | 33(66.0%) |
| Female | 17(34.0%) |
| Follow-up time |  |
| range | 9-144(month) |
| mean | 44.7（month） |
| LDH |  |
| Normal | 29(58.0%) |
| Elevated | 21(42.0%) |
| ECOG |  |
| 0 | 29(58.0%) |
| 1 | 18(36.0%) |
| 2 | 3(6.0%) |
| IPI score |  |
| 0-1 | 33(66.0%) |
| 2 | 10(20.0%) |
| 3 | 7(14.0%) |
| Ann Arbor Stage |  |
| I | 17(34.0%) |
| II | 23(46.0%) |
| III | 4(8.0%) |
| IV | 6(12.0%) |
| Surgery |  |
| Yes | 43(86.0%) |
| No | 7(14.0%) |
| Chemotherapy |  |
| Yes | 49(98.0%) |
| No | 1(2.0%) |
| Risk group |  |
| low | 38(78.0%) |
| intermediate | 2(4.0%) |
| high | 10(20.0%) |


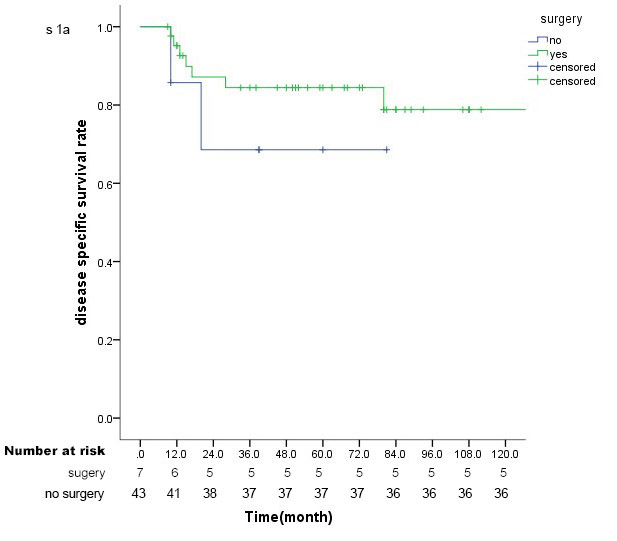


Supplement Figure 1a The 5 year CSS of patients in the surgical group was 78.8%, while the 5-year CSS of patients in conservative treatment group was 68.6%.


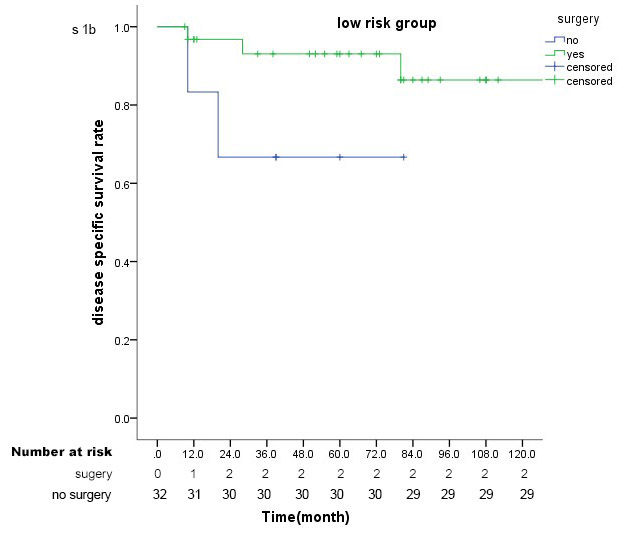


Supplement Figure 1b The 5 year CSS of patients in the surgical group was 86.4%, while the 5-year CSS of patients in conservative treatment group was 66.7% in low risk group.
